# Embalming, Viewing and the Social Construction of the Corpse: Time for Another Look

**DOI:** 10.1093/geroni/igab046.2853

**Published:** 2021-12-17

**Authors:** Redmond Finney, Lisa Shulman, Raya Kheirbek

**Affiliations:** 1 University of Maryland School of Medicine, Baltimore, Maryland, United States; 2 University of Maryland School of Medicine, University of Maryland School of Medicine, Maryland, United States

## Abstract

Embalming of the dead is more common in the United States than anywhere else in the world. Battles far from home during the Civil War with concern for contagion from dead bodies being shipped home, compelled President Lincoln to direct the troops to use embalming to allow the return of the Union dead to their homes. Viewings were common with war heroes and culminated with the viewing of Lincoln himself. In the 20th century embalming became a tradition despite substantial evidence indicating environmental and occupational hazards related to embalming fluids and carbon dioxide generated from manufacturing steel coffins before placing in concrete burial vaults. Embalming is promoted and considered helpful to the grieving process when families are comforted by a the appearance of a peaceful death. Embalmers are expected to produce an illusion of rest, an image that in some ways disguises death for the benefit of mourners. The dead are carefully displayed in a condition of liminal repose where the 'true' condition is hidden, and death is removed from the actual event. In this paper we highlight the spiritual and cultural complexities of embalming related- issues. We also provide data on the lack of grieving families’ preparedness for the financial burden associated with the death of a loved one and the lack of knowledge of alternative options. We propose an innovative process to empower people facing serious illness, and their families to make shared and informed decisions, especially when death is the expected outcome.

